# Mushroom and Kefir Functional Characterizations: Hypolipidemia and Gut Microbiota Modulations in Rat Models

**DOI:** 10.1002/fsn3.4503

**Published:** 2024-10-31

**Authors:** Huda Aljumayi, Amani A. Alrasheedi, Thamer Aljutaily, Isam A. Mohamed Ahmed, Nazeha A. Khalil

**Affiliations:** ^1^ Department of Food Science and Nutrition, College of Sciences Taif University Taif Saudi Arabia; ^2^ Department of Food and Nutrition, Faculty of Human Sciences and Design King Abdulaziz University Jeddah Saudi Arabia; ^3^ Department of Food Science and Human Nutrition, College of Agriculture and Food Qassim University Buraydah Saudi Arabia; ^4^ Department of Food Science and Nutrition, College of Food and Agricultural Sciences King Saud University Riyadh Saudi Arabia; ^5^ Department of Food Science and Technology, Faculty of Agriculture University of Khartoum Shambat Sudan; ^6^ Nutrition and Food Sciences Department, Faculty of Home Economics Menoufia University Shibin el Kom Egypt

**Keywords:** atherogenic indexes, high‐fat diets, lipid profile and functional foods, probiotic and prebiotics, short‐chain fatty acids

## Abstract

Hyperlipidemia is a malnutrition disease associated with different lifestyle factors mainly high fat/cholesterol foods consumption and less physical activity. Consumption of high fiber foods (prebiotic sources) additionally to gut microbiota (GM; probiotics species) could overcome hyperlipidemia and its associated risks. Prebiotics and probiotics are known by protective effects in different diseases like diabetes, inflammatory bowel diseases, and cardiovascular diseases. Mushroom and kefir milk (KM) are known for their high pre/probiotic nutritional values depending on many factors, for example, eating levels and/or conditions. Therefore, this study aimed to measure the potential health benefits of dried powdered mushrooms (DPM) supplemented with KM between hyperlipidemia rats in association with lipid profile, atherogenic index (AI), and GM profile. Rats were randomly divided into main negative control healthy group (G1; −ve), positive control hyperlipidemia (G2; +ve) and three hyperlipidemia groups (G3:G5) fed DPM at 2.5%, 5%, and 10% of rats' diet additionally to 5% KM (DPM + 5% KM) each, respectively. The collected blood samples used for glucose, lipid profile (cholesterol, triglycerides, high‐density lipoprotein cholesterol, and low‐density lipoprotein cholesterol), and AI in addition to fecal sample for GM and short‐chain fatty acids (SCFAs). Collected data illustrated that body weight, blood glucose, lipid profile, GM (*Bifidobacteria* and *Lactobacillus* vs. *Clostridium histolyticum*), AI, and SCFAs were improved between hyperlipidemia fed 5% both PDM + KM (*p* ≤ 0.05) at the best levels. In conclusion, same DPM/KM levels have broad development as functional active foods that could lower hyperlipidemia incidence and promotes intestinal health; however, much more human studies are needed.

## Graphical abstract

This study measures the potential health benefits of dried powdered mushrooms (DPM) supplemented with kefir milk (KM) between hyperlipidemia rats in association with lipid profile, atherogenic index (AI), and GM profile. The results illustrated that body weight (BW), blood glucose (BG), lipid profile, GM (*Bifidobacteria* and *Lactobacillus* vs. *Clostridium histolyticum*), AI, and SCFAs were improved between hyperlipidemia fed 5% both PDM + KM (*p* ≤ 0.05) at the best levels. DPM and KM supplements have broad development as functional active foods that could lower hyperlipidemia incidence and promote intestinal‐health.

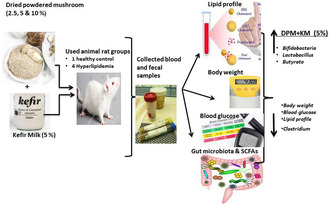

## 1 Introduction

Nutrition is important in human health and diseases while the malnutrition concept is known by unhealthy and unbalanced diets either by any deficiencies and/or any excesses in energy and/or nutrient intake as in many diseases such as diabetes, inflammatory bowel diseases, and cardiovascular diseases (CVDs) with their risk contributors (Khalil 2022; Hijová 2023). Hyperlipidemia is a risk contribution factor for CVDs by its induced oxidative stress causing endothelial dysfunction which can be observed by lipid peroxide levels in addition to micro/macro‐vascular damage associated with dyslipidemia. The measured atherogenic index (AI) is a good tool to measure such damage and that consists of triglycerides (TG) and high‐density lipoprotein cholesterol (HDL‐c) (Zhu et al. 2018). The CVDs and hyperlipidemia both are associated with different nutrients in dietary intake mainly the high fat/cholesterol dietary foods (F/CDFs) in contrast to high antioxidant dietary sources and low‐fat diets known by their protective effects in addition to the different cooking and/or preservation methods (Liu, Zhong, and Li 2023; Pavlidou et al. 2022). Such unhealthy conditions have been associated with the traditional Western dietary pattern that is identified by high levels of animal products and processed foods in contrast to low fruits and vegetables consumed levels, exacerbating to elevate the CVDs along with their risk problems. So lifestyle changes or medications, especially within dietary modifications (high fruit and vegetable consumption with low dietary fat levels), may help to prevent life‐threatening heart problems such as CVDs and hyperlipidemia.

Edible mushrooms and many vegetables are well known for their nutritional and medical values because of great amounts of protein, total phenolic compounds, and antioxidant activities in addition to being excellent sources of valuable vitamins and minerals. It is also a good source of different vitamins such as B‐group vitamins especially B9 (Folate or folic acid) and B12 (cyanocobalamin) additionally to V.C, V.A, and V.D (Ruiz‐Almenara, Gándara, and Gómez‐Hernández 2019; Bell et al. 2022).

The good levels of different minerals are again presented: phosphorus, potassium, iron, and copper (Mustafa et al. 2022). Moreover, another health issue with mushrooms is that it is low in calories in addition to being described as a potent herbal medicine because of their levels of important phytochemicals that support many aspects of benedictional health pharmacological activities such as antioxidants, anticancer, antiaging, and hyperlipidemia that in total is important for reducing many human health problems, especially the chances of having CVDs in addition to boost the immune system. Depending on all such health aspects, mushroom could be counted as one of the best prebiotic dietary sources that is well known as special healthy compounds (mainly plant fibers) acting as foods for human gut microbiota (GM) and being used for improving the balance and/or foster the growth or activity of beneficial GM (Liu, Zhong, and Li 2023). Indeed mushrooms show prebiotic activity that beneficially affects gut homeostasis performance in addition to the balance of GM that has been enhanced, supporting health and promising therapeutic agents (Fokunang et al. 2022; Bell et al. 2022). The first prebiotic concept was established as “non‐digestible food ingredient that beneficially affects the host by selectively stimulating the growth and/or activity of one or a limited number of bacteria already resident in the colon” in 1995 (Gibson and Roberfroid 1995).

On the other hand, probiotics have been recognized as special bacteria‐containing foods with beneficial potential effects in order to improve and help human health so prebiotics could be recognized as the supply of the existing probiotic bacteria with the fuel they need for a healthy human gut (Hijová 2023; Mayta‐Tovalino et al. 2023). Such a healthy human gut especially with intestinal probiotics species has been shown to contribute to regulating energy balance and cardiovascular benefits in addition to improving the intestinal barrier function with pro‐inflammatory cytokines reductions (Mayta‐Tovalino et al. 2023). On the other hand, the alternated GM known as dysbiosis results in an imbalanced intestinal ecosystem leading to different human health problems such as obesity, diabetic conditions, and CVDs in contrast to a healthy gut (Million et al. 2013; Valdes et al. 2018). Several intervention studies illustrated the significant health impacts of certain beneficial probiotics oral administration such as Firmicutes, Actinobacteria, *Lactobacilli*, and *Bifidobacterium* on body weight (BW) suggesting a correlation between GM and body fat regulation (Mayta‐Tovalino et al. 2023; Gibson et al. 2017). Therefore, mushrooms are t recomended to be a regular part of the human diet as a prebiotic dietary source because of their benefit in preventing or delaying different many human diseases. For example, mushrooms serve as natural potential gift for human health with different therapeutic effects and activities with many diseases such as anti‐diabetic, antibacterial, immune‐stimulatory properties that considered mushroom previously as “elixir of life.” However, such prebiotic and nutritional effects in mushrooms vary depending on preparation and/or cooking methods/forms (fresh, boiled, microwaved, steamed, dried, puree, paneer, and pulaw in addition to soup; each has its own advantages and disadvantages). Indeed mushrooms as many boiling vegetables that are well known for removing up to 90% of main nutrients, so it is recommended to be prepared in different ways such as roast, steam, stir‐fry, microwave, and/or dried form.

Additionally, uncooked vegetables reported previously to bind the bile acid that has been associated with cholesterol (CHO) levels as it can alter the recirculation of bile acids in order to affect the CHO utilization and fat absorption; in total could be attributed to different heart problems. Additionally, fat accumulation again in the liver has been linked with the AIs estimation that is a prediction of artery sclerosis (Hijová 2023). Moreover, the probiotics dietary sources established recently showed significant total CHO and low‐density lipoprotein cholesterol (LDL‐c) reduction, especially within hypercholesterolemia subjects. So dietary probiotics supplementations in association with prebiotics dietary sources (known as synbiotics) are preferred to confer most human health benefits such as protections and/or potential therapy for CVDs by CHO levels and oxidative stress reductions (Liu, Zhong, and Li 2023). Such synbiotics (probiotics and prebiotics combination) is the subject of extensive recent research to balance the functional and structural changes or in an effective way to alter GM compositions and activities. Indeed, synbiotics supplementation, especially by *Lactobacillus casei*, *Lactobacillus rhamnosus*, *Streptococcus thermophilus*, *Bifidobacterium breve*, *Lactobacillus acidophilus*, *Bifidobacterium longum*, *Lactobacillus bulgaricus*, and fructooligosaccharides between metabolic syndrome (MetS) patients, reduced their body mass index (BMI) and fasting blood glucose (BG) levels (Pavlidou et al. 2022; Rabiei et al. 2019; Tenore et al. 2019). The main GM activities are the functional metabolites or metabolic end products. Short‐chain fatty acids (SCFAs) and bile acids are negatively metabolic products associated with cardiometabolic diseases and affect human health positively (Hijová 2023). Indeed the previous published data from our group demonstrated positive effects of symbiotic supplementations between diabetic animal models in both GM compositions and activities (Khalil et al. 2021). Such a study was established by using Arabic Gum and yogurt supplementations in a good correlated effect of consumed probiotic and prebiotic dietary sources. Also, yogurt used as probiotics in the model's diet could help bind to bile acids and increase their excretion at the same time and reduce the bile acid recycling in the enterohepatic circulation system. However, fermented kefir dairy milk products similar to yogurt in many ways have not been used in association with GM levels between hyperlipidemia models. It is rich in calcium, protein, and vitamin B levels in addition to containing high probiotics unique species such as *Lactobacillus kefiri* that are able to support and boost digestive health. It can also manage blood sugar levels with CHO reduction (Bellikci‐Koyu et al. 2019).

Therefore, prebiotics and probiotics dietary modification has been suggested in the current study as a useful strategy for improving the health status via GM modulation. Thus the aim of the current study was to evaluate the effects of supplemented probiotic and prebiotic dietary sources on GM composition levels between hyperlipidemia animal models. Dried powdered mushroom (DPM) supplemented samples (DPM at different levels: 2.5%, 5%, and 10%) in addition to 5% kefir milk (KM) have been used between hyperlipidemia animal models compared to the control negative healthy and control hyperlipidemia positive groups. BW have been recorded and calculated before and after running the experiment, and then the collected serum blood samples at the end of the experiment have been used for different analyses (glucose, lipid profile, and atherogenic indices) in addition to GM compositions and activities from fecal samples.

## 2 Materials and Methods

### 2.1 Materials

#### 2.1.1 Tested Substance Collections and/or Preparations

Fresh white mushroom (*Agaricus bisporus*) samples were obtained from the Agricultural Research Center, especially at the Horticulture Research Institute, Ministry of Agriculture, Giza, Egypt. They were dried at 40°C and powdered before being stored at 4°C and then used for the procedures as DPM along with low‐fat KM that was bought from the local market (used at 5%; Khalil et al. 2021).

#### 2.1.2 Chemical Materials Collection

All used chemical supplements were acquired from a local medical company in the city of Cairo, Egypt (Al‐Gomhouria Pharmaceutical Company).

### 2.2 Methods

#### 2.2.1 Mushroom Samples Preparation

All white mushroom (*A. bisporus*) samples were cleaned under a running water tap, cut into slight pieces, and dried at 40°C as described by other previously published data with date fibers within some modifications (Khalil et al. 2023). Then all the dried samples were powdered, and DPM were mixed randomly before being used directly to the animal's diet in three different concentrations of 2.5%, 5%, and 10% as in the following used procedures in combination with 5% KM supplementations.

#### 2.2.2 Used Animal Model

Forty male albino rats (110–120 g) were obtained from the National Training Center, Cairo, Experimental Animal Care Centre, and Egypt. All rats were housed at the Faculty of Home Economics, Menoufia University, Egypt, after obtaining ethical approval (16‐SREC‐04‐2023), and they were adapted in a week time by feeding the control normal diet formulated as described by our published previous data (Khalil et al. 2024), according to the American Institute of Nutrition recommendation for rodent growth (Reeves, Nielsen, and Fahey 1993). The basil (control) diet composition could be found as supporting information in our previous papers (Aljutaily et al. 2022), ingredients (g/100 g diet), vitamin, and mineral mixtures (g/1000 g). Finally all rats were divided randomly following the experimental design.

#### 2.2.3 Experimental Animal Model Design

All rats were divided randomly into five groups (*n* = 8 each); four animal groups (G2:G5) fed high‐fat diets for 6 weeks (hyperlipidemia induction; HFD) as described by our previous published studies (Aljutaily et al. 2022; Li et al. 2022) while one group fed control normal diet to be used as a negative control group (G1; −ve). Three hyperlipidemia groups out of the four received water and diet up to 6 weeks; the control positive hyperlipidemia group (G2; +ve) in addition to G3, G4, and G5 by 2.5%, 5%, and 10% of DPM was supplemented with 5% KM for about a month (28 days) each, respectively. All the animal BW was recorded before and after running the experiment and then the changed BW levels were calculated. Also, collected serum blood samples at the end of the experimental were kept at −80°C until further analysis.

#### 2.2.4 BW Monitoring and Calculation

All the animal BW was recorded before and after running the experiment, the change in the animal BW was calculated as the differences between the final and intimal BW, and then all the groups' BW levels were compared to the control positive hyperlipidemia group using the formula: (each group final body weight − control positive final body weight group)/control positive final body weight group × 100 (Chapman, Castillo, and Campbell 1959).

#### 2.2.5 Collected Serum Blood Samples

At the end of the experiment, the rats were sacrificed and blood samples from the abdominal aorta were collected and kept at room temperature for 30 min. All blood collected samples were then centrifuged at 3000 rpm for 10 min to collect the serum samples that were carefully aspirated and then transferred into clean cuvette tubes and kept at −20°C until further biological analysis.

#### 2.2.6 Biochemical Analysis

Serum glucose, lipid profile, and GM compositions were determined by following analytical procedures described according to the method of Khalil et al. (2021) and Rojas et al. (1999), respectively, for GM compositions and serum glucose while the serum total CHO was carried out according to Allain et al. (1974). Also serum TG were determined by enzymatic method using kits according to Fossati and Prencipe (1982) while the HDL‐c was established according to the method described by Burstein, Scholnick, and Morfin (1970) and very low‐density lipoprotein cholesterol (vLDL‐c) was calculated in mg/dL according to Niemann and Lee (1996) using the following formula: vLDL‐c (mg/dL) = TG/5. Additionally, LDL‐c was calculated in mg/dl as follows: LDL‐c (mg/dL) = Total CHO − (HDL‐c + vLDL‐c). Regarding the colonic microbiota determination, it was carried out according to our previous published data (Khalil et al. 2021). The AIs were calculated as levels of collected CHO/HDL‐c levels and also, levels of LDL‐c/HDL‐c in addition to atherogenic fraction (AF) that was calculated by the differences between TC and HDL‐c as that has been established within the recent published study from our group (Khalil, Alfaris, and Altamimi 2022).

#### 2.2.7 Gut Microbiome Collection and Evaluations

Regarding the colonic microbiota determination (GM), fecal samples collection was obtained from all used animal models after 6‐week hyperlipidemic inductions (called the start point), and then it was collected again at the end of the treatment period (nearly a month and called end point). They were used for GM compositions after being prepared, homogenized, and vortexed as described within our previous published data, and the counted bacterial genera were measured for *Bifidobacteria*, *Clostridium histolyticum*, and *Lactobacillus* in addition to the total bacterial numbers by fluorescence in situ hybridization (FISH; Khalil et al. 2014) that expressed as log_10_ CFU g^−1^.

#### 2.2.8 SCFAs Determination

The produced SCFAs levels (0‐h/start and final stages) also after 6‐week hyperlipidemic inductions (called the start point) then it has been collected again at the end of the treatment period (nearly a month and called end point) then all samples were analyzed as described within our published data (Khalil et al. 2014) by performed gas–liquid chromatography (GC) method with an HP 5890 series II GC system (Hewlett Packard, Palo Alto, California) that was equipped with a capillary fused silica‐packed column (Permabond FFAP J&W Scientific). The elution times were recorded (acetic acids, propionic acid, and butyric acid) and then the data were analyzed.

#### 2.2.9 Histology Structure Examinations

The current study used collected samples from the experimental organs (kidney) for histological structure evaluations that all were used for measuring the effects of different dried powdered mushrooms and kefir milk (DPM/KM) supplementations between hyperlipidemia rat models. All the experimental animals were sacrificed and the collected kidney samples were fixed in neutral buffered formalin (10%), and later they were routinely processed and embedded in paraffin wax after being dehydrated in alcohol and cleared in xylol. Then all prepared paraffin blocks were sectioned at 4–5 μm thickness and were also stained with hematoxylin and eosin (H&E) as described within our previous published data (Khalil et al. 2021).

#### 2.2.10 Statistical Analysis

The data are expressed as the mean ± standard deviation (SD). Differences between the groups were analyzed using one‐way analysis of variance (ANOVA). Differences between pairs of means were subsequently tested using Duncan's multiple range as a post hoc test. The statistical analysis was carried out using SPSS version 21.0 software (SPSS Inc., Chicago, IL, USA). Data were considered statistically significant differences at *p* ≤ 0.05.

## 3 Results

All collected data have been illustrated and presented within the following sections after being statistically analyzed.

### 3.1 The Effect of DPM/KM Supplementations on BW Between Hyperlipidemia Animal Models

Animal models in the current study have been used to measure the effects of different DPM in addition to low‐fat KM consumption on the BW changes (Table 1).

**TABLE 1:** Changes in body weight gain of dried powdered mushrooms and kefir milk (DPM/KM) supplementations between hyperlipidemia rats.

Groups	Initial body weight (g)	Final body weight (g)	Differences (initial − final; g)	Changes from control (+; g)
G1: Negative control (−ve)	118.56 ± 1.51^a^	330.22 ± 3.20^a^	+211.66	−33.34
G2: Positive control (+ve)	117.32 ± 1.51^a^	363.56 ± 2.50^d^	+246.24	—
G3: Fed 2.5 DPM + KM	117.60 ± 1.14^a^	350.20 ± 1.72^c^	+232.60	−13.36
G4: Fed 5 DPM + KM	118.23 ± 1.51^a^	330.23 ± 1.01^a^	+212.00	−33.33
G5: Fed 10 DPM + KM	121.25 ± 2.63^a^	343.10 ± 3.59^b^	+221.85	−20.46

*Note:* Data represent mean ± SD (*n* = 8). Means in the same column with different superscript letters (a, b, c and d) are significantly different (*p ≤ 0.05*).

Abbreviations: DPM, dried powdered mushroom; KM, kefir milk supplementations (all at 5% each).

Table 1 illustrates the collected BW data and shows that all the animal models used within the start time point (intimal BW) are nearly at the same BW levels (about 118 g). However, at the end of the experiment, all the rat groups got different BW levels; all the animals' BW has been increased. The biggest BW levels between all the groups have been obtained with the positive control hyperlipidemia rats group significantly (363.56 ± 2.50 g; *p* ≤ 0.05). Such BW levels have been increased by +246.00 g from the start time point (Table 1). Additionally, the second animal model with their final BW levels has been verified with the rats group fed 2.5 DPM + 5% KM (350.20 ± 1.72) that have been increased by about +233 g BW significantly (*p* ≤ 0.05) and that was followed by the rats fed 10 DPM + 5% KM and then the rats fed 5 DPM + 5% KM by about +221.85 and +212.00 g, respectively.

Regarding the changes in BW levels from the control positive rats group, Table 1 again illustrates that both animal groups fed mixed mushroom and KM at the same levels (5 DPM + 5% KM; 5% each) and the negative control group got similar effects on the rats BW (decreased by about 33 g). Again rats consumed 10 DPM + 5% KM presented reduction within their final BW by about 20 g while the group rats fed 2.5 DPM + 5% KM showed the lowest declined BW levels (about 14 g). To conclude up, both supplemented dried mushroom powder (DPM) and KM in the same levels illustrated the best effective treatment on the BW change levels between the hyperlipidemia used animal models.

### 3.2 The Effect of DPM/KM Supplementations on Serum Glucose Levels Between Hyperlipidemia Animal Models

The collected serum glucose levels obtained from the hyperlipidemia rats are shown in Table 2. It can be noticed that all the rat groups were with low serum glucose levels significantly (*p* ≤ 0.05) except the group fed 2.5 DPM + 5% KM that presented significant serum glucose levels (143.29 ± 3.78 mg/dL; *p* ≤ 0.05). The lowest levels were seen with the animal model fed same levels of both DPM and KM (5% each; 105.31 ± 1.15 mg/dL) and that was close to the negative control healthy rats fed basal diet (101.76 ± 2.08 mg/dL; Table 2). The second low rat group with serum glucose levels was the animal models that consumed 10 DPM + 5% KM at 118.02 ± 2.64 mg/dL. Finally the largest serum glucose levels were gotten with the group served with 2.5 DPM + 5% KM (143.29 ± 3.78 mg/dL). Table 2 again illustrates the relative changes of BG levels between all rat's group to the control positive hyperlipidemia group; it can be noticed from Table 2 that glucose levels have been declined between all the animal models used in the current experiment compared to the positive hyperlipidemia group levels that show the biggest glucose level (157.67 ± 1.52 mg/dL). Moreover, the rats fed 5% of each supplemented dietary sources (DPM + KM) had the lowest glucose levels significantly compared to all the hyperlipidemia groups, which decreased significantly from the positive group by −52.36 mg/dL (*p* ≤ 0.05). While such group was similar to the control negative rats group's (−ve; 101.76 ± 2.08 mg/dL) glucose levels. Additionally, the same group was followed by the reduction of serum glucose levels of animal model consumed 10 DPM + 5% KM at 118.02 ± 2.64 mg/dL which was decreased by about 52 mg/dL glucose levels and finally followed by the rats group fed 2.5 DPM + 5% KM at about 14 mg/dL glucose reduction significant levels (Table 2; *p* ≤ 0.05).

**TABLE 2:** Changes in blood glucose levels of dried powdered mushrooms and kefir milk (DPM/KM) supplementations between hyperlipidemia rats.

Groups	Glucose level (mg/dL)	Relative change of control (+; mg/dL)	% Changes of control (+)
G1: Negative control (−ve)	101.76 ± 2.08^a^	−55.91	−35.46
G2: Positive control (+ve)	157.67 ± 1.52^d^	—	—
G3: Fed 2.5 DPM + KM	143.29 ± 3.78^c^	−14.38	−09.12
G4: Fed 5 DPM + KM	105.31 ± 1.15^a^	−52.36	−33.20
G5: Fed 10 DPM + KM	118.02 ± 2.64^b^	−39.65	−25.14

*Note:* Data represent mean ± SD (*n* = 8). Means in the same column with different superscript letters (a, b, c and d) are significantly different at (*p* ≤ 0.05).

Abbreviations: DPM, dried powdered mushroom; KM, kefir milk supplementations (all at 5% each).

### 3.3 The Effect of DPM/KM Supplementations on Lipids Profile Levels Between Hyperlipidemia Animal Models

The presented data in Table 3 revealed the measured lipid profile levels, especially for CHO, TG, HDL‐c, LDL‐c, and vLDL‐c between used animal models. It can be noticed that the biggest significant CHO levels were seen in hyperlipidemia rat group that consumed mixtures of 10 DPM + 5% KM (group G5; 98.37 ± 1.10 mg/dL). It has been followed significantly as shown again in Table 3 by the hyperlipidemia rat group rats of G3 and G4 consumed 2.5 DPM + KM and 5 DPM + KM, respectively, with the same 5% KM supplemented levels each (88.17 ± 1.96 and 64.03 ± 1.40 mg/dL respectively; *p* ≤ 0.05). Such G4 was the nearest CHO levels to the control negative group (G1: fed normal healthy basal diet) that reached 63.43 ± 1.65 mg/dL (Table 3).

**TABLE 3:** Changes in lipid profile measured of dried powdered mushrooms and kefir milk (DPM/KM) supplementations between hyperlipidemia rats.

Groups	Lipid profile (mg/dL)				
	CHO	TG	HDL‐c	LDL‐c	vLDL‐c
G1: Negative control (−ve)	63.43 ± 1.65^a^	24.17 ± 3.21^a^	56.14 ± 1.11^d^	2.46 ± 0.49^a^	4.83 ± 0.51^a^
G2: Positive control (+ve)	107.37 ± 0.36^e^	122.40 ± 2.02^e^	18.35 ± 2.48^a^	64.53 ± 2.75^e^	24.48 ± 0.30^e^
G3: Fed 2.5 DPM + KM	88.17 ± 1.96^c^	70.40 ± 1.10^c^	41.19 ± 3.40^b^	32.90 ± 2.83^d^	14.08 ± 0.11^c^
G4: Fed 5 DPM + KM	64.03 ± 1.40^b^	41.13 ± 2.41^b^	43.91 ± 1.12^c^	11.90 ± 0.30^b^	8.23 ± 0.21^b^
G5: Fed 10 DPM + KM	98.37 ± 1.10^d^	81.40 ± 0.47^d^	61.38 ± 1.12^e^	20.70 ± 1.20^c^	16.28 ± 0.27^d^

*Note:* Data represent mean ± SD, values are not sharing superscript letters (a, b, c, d and e) in the same column are significantly different (*p* ≤ 0.05).

Abbreviations: DPM, dried powdered mushroom; KM, kefir milk supplementations (all at 5% each).

Regarding the TG levels (Table 3), it has been illustrated that the largest significant TG levels have been seen within hyperlipidemia rats group (G5) that fed 10% DPM in addition to KM at 5% compared to the treated animal groups (81.40 ± 0.47 mg/dL; *p* ≤ 0.05). While the lowest significant hyperlipidemia group in TG obtained levels between treated groups have been seen with animal models fed 5% of both dried mushroom and KM (G4; 41.13 ± 2.41 mg/dL). Such collected data in Table 3 within the treated hyperlipidemia animal models are in comparison to both negative and positive control groups significantly (G1 and G2; 24.17 ± 3.21 and 122.40 ± 2.02 mg/dL, respectively). Additionally HDL‐c levels represented the highest significant levels in G5 hyperlipidemia group followed by fed G5: fed 10 DPM + KM, then G4 fed 5 DPM + KM, and finally G3 that consumed 2.5 DPM + KM (61.38 ± 1.12, 43.91 ± 1.12 and 41.19 ± 3.40 mg/dL, respectively). While the control negative and positive groups were significantly at 56.14 ± 1.11 and 18.35 ± 2.48 mg/dL, respectively, to all treated groups (Table 3). On the other hand, the measured LDL‐c levels presented in Table 3 as well were with its biggest significant levels at hyperlipidemia animal model group (G3) consumed 2.5 DPM + KM (32.90 ± 2.83 mg/dL; *p* ≤ 0.05) and that was the closest to the positive control group (G2; 64.53 ± 2.75 mg/dL). While the hyperlipidemia rats group (G4) consumed 5% of both mixed DPM and KM which shows the lowest significant LDL‐c levels (11.90 ± 0.30 mg/dL) compared to all the treated groups followed by G5 (rats fed 10% of DPM and 5% of KM; 20.70 ± 1.20 mg/dL).

Finally the vLDL‐c measurements shown in Table 3 between all used animal models were significantly in the highest significant amount between G5, the hyperlipidemia rats group consumed 10 DPM in addition to 5% KM supplementations (16.28 ± 0.27 mg/dL; *p* ≤ 0.05) and such vLDL‐c levels are too close to the negative healthy animal model rats fed basal diet (24.48 ± 0.30 mg/dL). Also the hyperlipidemia G3 rat groups fed 2.5 DPM + KM followed by G4 hyperlipidemia rats fed 5 DPM + KM were in big vLDL‐c levels compared to the positive hyperlipidemia rats group (14.08 ± 0.11 and 8.23 ± 0.21 vs. 24.48 ± 0.30 mg/dL, respectively). To conclude up, all lipid profile levels improved between the used hyperlipidemia animal models, especially between rats fed 5% of both DPM in combinations to 5% KM consumption for all measured lipid parameters.

### 3.4 The Effect of DPM/KM Supplementations on AI Levels Between Hyperlipidemia Animal Models

Table 4 illustrates the effect of used DPM in addition to KM (DPM/KM) on the AI levels between the used hyperlipidemia rat models.

**TABLE 4:** Changes in atherogenic index levels of dried powdered mushrooms and kefir milk (DPM/KM) supplementations between hyperlipidemia rats.

Groups	Atherogenic measured parameters (mg/dL)		
	Cholesterol/HDL‐c	LDL‐c/HDL‐c	Cholesterol‐HDL‐c
G1: Negative control (−ve)	1.13 ± 0.01^a^	0.04 ± 0.00^a^	7.29 ± 0.61^a^
G2: Positive control (+ve)	5.85 ± 0.97^c^	3.52 ± 0.73^c^	89.01 ± 3.13^e^
G3: Fed 2.5 DPM + KM	2.14 ± 0.16^b^	0.80 ± 0.14^b^	46.98 ± 2.63^d^
G4: Fed 5 DPM + KM	1.46 ± 0.01^ab^	0.27 ± 0.01^ab^	20.13 ± 0.33^b^
G5: Fed 10 DPM + KM	1.60 ± 0.03^ab^	0.34 ± 0.02^b^	36.99 ± 1.13^c^

*Note:* Data represent mean ± SD (*n* = 8). Means in the same column with different superscript letters (a, b, c, d and e) are significantly different at (*p* ≤ 0.05).

Abbreviations: DPM, dried powdered mushroom; KM, kefir milk supplementations (all at 5% each).

The CHO to HDL‐c rational showed the lowest levels between the normal healthy control negative group (G1; −ve) and that reached 1.13 ± 0.01 mg/dL. However, the hyperlipidemia rats in the positive control group (G2; +ve) presented the biggest significantly different CHO to HDL‐c rational levels (5.85 ± 0.97 mg/dL; *p* ≤ 0.05). Additionally, the treated hyperlipidemia animal rats were at the lowest CHO to HDL‐c levels when they fed on 5% of both supplemented DPM and KM (G4; 1.46 ± 0.01 mg/dL). Such rats group was followed by the rats on G5 and that was fed 10% of DPM and 5% of supplemented KM (CHO to HDL‐c was 1.60 ± 0.03 mg/dL). Finally, the CHO to HDL‐c rational levels were at the top significantly different between hyperlipidemia rats fed 2.5 DPM and 5% of KM additions (2.14 ± 0.16 mg/dL; *p* ≤ 0.05). Again, the levels of LDL‐c/HDL‐c are illustrated in Table 4 and are at the lowest significant levels at 0.04 ± 0.00 mg/dL between the healthy control negative group (−ve; *p* ≤ 0.05). On the other hand the positive hyperlipidemia rats show the highest LDL‐c/HDL‐c levels that reached 3.52 ± 0.73 mg/dL. While all hyperlipidemia rat groups fed different supplemented dietary mixtures were arranged as 0.27 ± 0.01 < 0.34 ± 0.02 < 0.80 ± 0.14 mg/dL and were presented as the best effective groups G4 < G5 < G3 that all fed both supplemented 5% KM in addition to DPM at 5%, 10%, and 2.5%, respectively, with best significantly difference effective levels at 5% of both DPM and KM (*p* ≤ 0.05).

Finally, an additional atherogenic measured parameter was for the levels of differences between CHO and HDL‐c as mg/dL. It is demonstrated in Table 4 that such difference levels were the biggest at the hyperlipidemia animal control rats (G2; +ve) that was in contrast to the negative control rats group (G1; −ve): 89.01 ± 3.13 versus 7.26 ± 0.61 mg/dL. Also, the differences between CHO and HDL‐c with hyperlipidemia rat group consumed 5% additions of both DPM and KM at good levels of 20.13 ± 0.33 mg/dL. Such differences between CHO and HDL‐c levels were followed significantly (*p* ≤ 0.05) by the rats group fed high levels of DPM in addition to the 5% KM (10% additions; 36.99 ± 1.13 mg/dL) while the last rats group was seen with the rats consumed 2.5% of DPM and 5% of KM additions; differences between CHO and HDL‐c levels were at 46.98 ± 2.63 mg/dL (significant difference; *p* ≤ 0.05).

### 3.5 The Effect of DPM/KM Supplementations on Gut Microbiota Composition Between Hyperlipidemia Models

**FIGURE 1:**
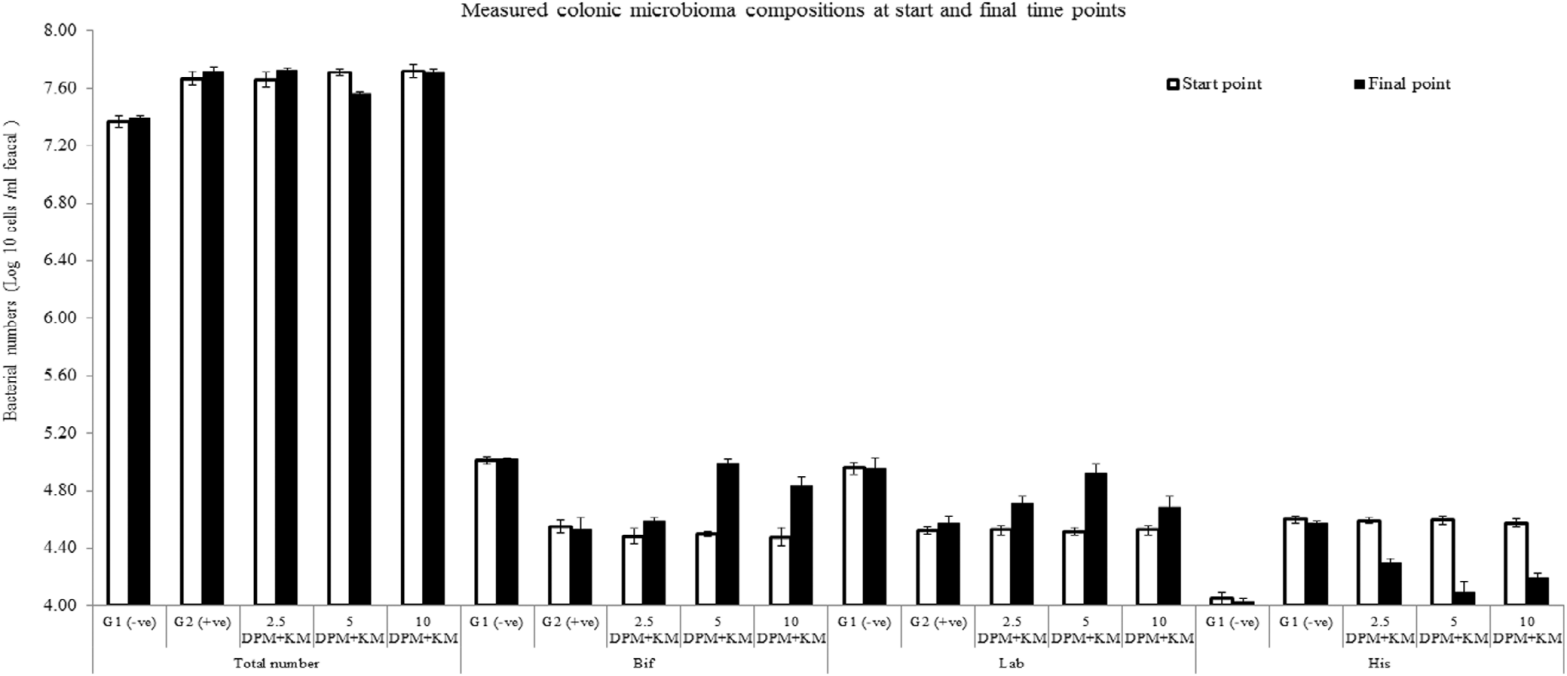
Changes in gut microbiota composition of dried powdered mushrooms and kefir milk (DPM/KM) supplementations between hyperlipidemia rats. G1 (−ve): Healthy normal rats fed with basal diet and without any DPM + KM additions; G2 (+ve): Hyperlipidemia rats fed with basal diets without any PDM + KM additions; the third group is 2.5 PDM + 5% KM supplemented in hyperlipidemia rats fed with basal diets; the 4th group is 5 PDM + 5% KM supplemented in hyperlipidemia rats fed with basal diets; and finally the last group fed 10 PDM + 5% KM supplemented in hyperlipidemia rats fed with basal diets.

The effect of using DPM in addition to KM (DPM/KM) has been illustrated on changes in GM between hyperlipidemia rat models (Table 5 and Figure 1). The measured counted bacterial genera were *Bifidobacteria*, *Lactobacillus*, and *Clostridium histolyticum* in addition to the total bacterial numbers. Firstly, the total bacterial measured numbered were the top numbers between all the experimental rat models. However, it can be seen from Table 5 and Figure 1 that the total bacterial number was at low levels between the healthy animal control group (G1; −ve) with significant difference (*p* ≤ 0.05) at both start and final time points and that reached significant difference 7.37 ± 0.53 and 7.39 ± 0.47 log_10_ cells per mL of fecal slurry, respectively (*p* ≤ 0.05). On the other hand, the biggest bacterial numbers were seen between the positive control rats group (G2; +ve) again at both start and end time points (7.67 ± 0.05 and 7.72 ± 0.03 log_10_ cells per mL of fecal slurry, respectively; Table 5 and Figure 1). The numbers over all between hyperlipidemia rat groups (G3, G4 and G5) were demonstrated in Table 5 which also show similar levels at the start point (7.66 ± 0.05, 7.71 ± 0.02 and 7.72 ± 0.05 log_10_ cells per mL of fecal slurry, respectively). While they all have changed significantly (*p* ≤ 0.05) at the end time points the best effective changes in the hyperlipidemia rats group fed both DPM and KM at 5% levels each in one mixture that is the nearest to the control healthy negative group (Table 5 and Figure 1; 7.56 ± 0.02 vs. 7.39 ± 0.47 log_10_ cells per mL of fecal slurry). It was followed by the other two hyperlipidemia rats groups (G4 > G5; 7.72 ± 0.02 > 7.71 ± 0.03 log_10_ cells per mL of fecal slurry).

**TABLE 5:** Changes in gut microbiota composition of dried powdered mushrooms and kefir milk (DPM/KM) supplementations between hyperlipidemia rats.

Groups	Time points	Bacterial counts (log_10_ cells per and fecal slurry)			
		Total number	*Bifidobacteria*	*Lactobacillus*	*Clostridium histolyticum*
G1: Negative control (−ve)	Start	7.37 ± 0.53^a^	5.01 ± 0.03^b^	4.96 ± 0.04^b^	4.05 ± 0.04^a^
	End	7.39 ± 0.47^A^	5.02 ± 0.01^C^	4.95 ± 0.07^C^	4.03 ± 0.03^A^
G2: Positive control (+ve)	Start	7.67 ± 0.05^b^	4.55 ± 0.04^a^	4.52 ± 0.03^a^	4.60 ± 0.03^b^
	End	7.72 ± 0.03^B^	4.53 ± 0.08^A^	4.57 ± 0.05^A^	4.57 ± 0.02^D^
G3: 2.5 DPM + KM	Start	7.66 ± 0.05^b^	4.49 ± 0.05^a^	4.53 ± 0.04^a^	4.59 ± 0.02^b^
	End	7.72 ± 0.02^B^	4.59 ± 0.03^A^	4.72 ± 0.05^B^	4.30 ± 0.03^C^
G4: 5 DPM + KM	Start	7.71 ± 0.02^b^	4.50 ± 0.02^a^	4.52 ± 0.03^a^	4.60 ± 0.03^b^
	End	7.56 ± 0.02^AB^	4.99 ± 0.03^C^	4.92 ± 0.06^C^	4.09 ± 0.08^A^
G5: 10 DPM + KM	Start	7.72 ± 0.05^b^	4.48 ± 0.07^a^	4.53 ± 0.03^a^	4.58 ± 0.03^b^
	End	7.71 ± 0.03^B^	4.84 ± 0.06^B^	4.68 ± 0.08^AB^	4.19 ± 0.03^B^

*Note:* Data represent mean ± SD (*n* = 8). Means in the same column with different superscript small letters (a and b) are significantly different at (*p* ≤ 0.05) within the start time points while means in the same column with different superscript capital letters (A, B, C and D) are significantly different (*p* ≤ 0.05) within the end time points.

Abbreviations: DPM, dried powdered mushroom; KM, kefir milk (5% each treatment).

Regarding the measured probiotic species in the current study, it can be seen in both Table 5 and Figure 1 that the numbers of *Bifidobacteria* and *Lactobacillus* were seen at similar levels with a slight increase in the *Bifidobacteria* numbers in all the experimental animal models. Moreover, the healthy control group presented significantly high *Bifidobacteria* numbers in start and end time points (nearly 5 log_10_ cells per mL of fecal slurry; *p* ≤ 0.05) rather than low numbers with all the other hyperlipidemia experimental animal models (approximately 4.5 log_10_ cells per mL of fecal slurry). Additionally, the most effective significant treated hyperlipidemia rats group with such bacterial probiotics species (*Bifidobacteria*; Table 5 and Figure 1) was seen with the hyperlipidemia rats fed on 5% DPM + MK mixture at the end of running the experimental (~4.99; nearly 5 log_10_ cells per mL of fecal slurry; *p* ≤ 0.05) that was nearly the same as seen with the control negative healthy rats group. Such collected *Bifidobacteria* numbers were followed at the end time point by the group rats supplemented by 10% PDM in 5% KM mixture and finally the 2.5% PDM and 5% KM mixture (4.84 ± 0.06 > 4.59 ± 0.03 log_10_ cells per mL of fecal slurry, respectively).

Moreover, the other measured probiotic species, *Lactobacillus* (Table 5 and Figure 1) numbers have been seen at the same levels as seen with the *Bifidobacteria* levels. The largest measured significant (*p* ≤ 0.05) numbers were seen with the healthy control rats at both the start and end of the experiment (about 4.95 log_10_ cells per mL of fecal slurry; Table 5 and Figure 1) in contrast to the unhealthy hyperlipidemia all groups (nearly the same numbers all at the start time point; 4.52 log_10_ cells *Lactobacillus* per mL of fecal slurry). While such measured species have been changed significantly at the end of the experimental of feeding PDM + KM mixtures with special respect to the rats group fed the mixture of 5% both PDM + KM (4.92 log_10_ cells *Lactobacillus* per mL of fecal slurry; *p* ≤ 0.05). Such *Lactobacillus* number was close to the numbers counted with the healthy control group (G1; −ve) at about 4.95 log_10_ cells *Lactobacillus* per mL of fecal slurry (Table 5 and Figure 1). Again the following rat groups with good *Lactobacillus* numbers at the end time points of running the experimental were seen with 10% PDM + KM additions and finally the 2.5% PDM + KM additions (4.68 then 4.72 log_10_ cells Lactobacillus per mL of fecal slurry; Table 5 and Figure 1).

On the other hand another intestinal microbial measured species was the *Clostridium histolyticum* (Table 5 and Figure 1). It can be seen that such species were all in the lowest significant numbers (4.05 log_10_ cells *Clostridium histolyticum* per mL of fecal slurry; Table 5 and Figure 1) within the control negative healthy group (G1; −ve) in contrast to all the hyperlipidemia rat groups (G2:G5), especially at the start time points (about 4.60 log_10_ cells *Clostridium histolyticum* per mL of fecal slurry; Table 5 and Figure 1). While the end time points show different significant numbers with each treated rats with especial respect to the group rats on G4 that fed 5% of both DPM + KM (mixture; *p* ≤ 0.05) and are the closest group to the control healthy group rats 4.09 ± 0.08 log_10_ cells *Clostridium histolyticum* per mL of fecal slurry (Table 5 & Figure 1). The second best treated rats group showed decreased *Clostridium histolyticum* numbers on 10% PDM + KM supplementations (4.19 ± 0.03 log_10_ cells *Clostridium histolyticum* per mL of fecal slurry; Table 5 and Figure 1). While the last significant effective mixture was seen with the 2.5% PDM + KM supplemented mixture at 4.30 ± 0.03 log_10_ cells *Clostridium histolyticum* per mL of fecal slurry; *p* ≤ 0.05.

To conclude, the best effective treatment used of PDM and KM has been recognized by increasing the probiotic calculated species (*Bifidobacteria* and *Lactobacillus*) in contrast to *Clostridium histolyticum* counted levels with respect to the mixture of 5% additions in both PDM and KM at the final time points. However, the lowest beneficial effective mixture was seen at 2.5% PDM and 5% KM additions that were followed finally by the 10% PDM with 5% KM mixture between all counted bacterial species that confirm such best prebiotic effects on such measured bacteria.

### 3.6 The Effect of DPM/KM Supplementations on SCFAs Between Hyperlipidemia Animal Models

**FIGURE 2:**
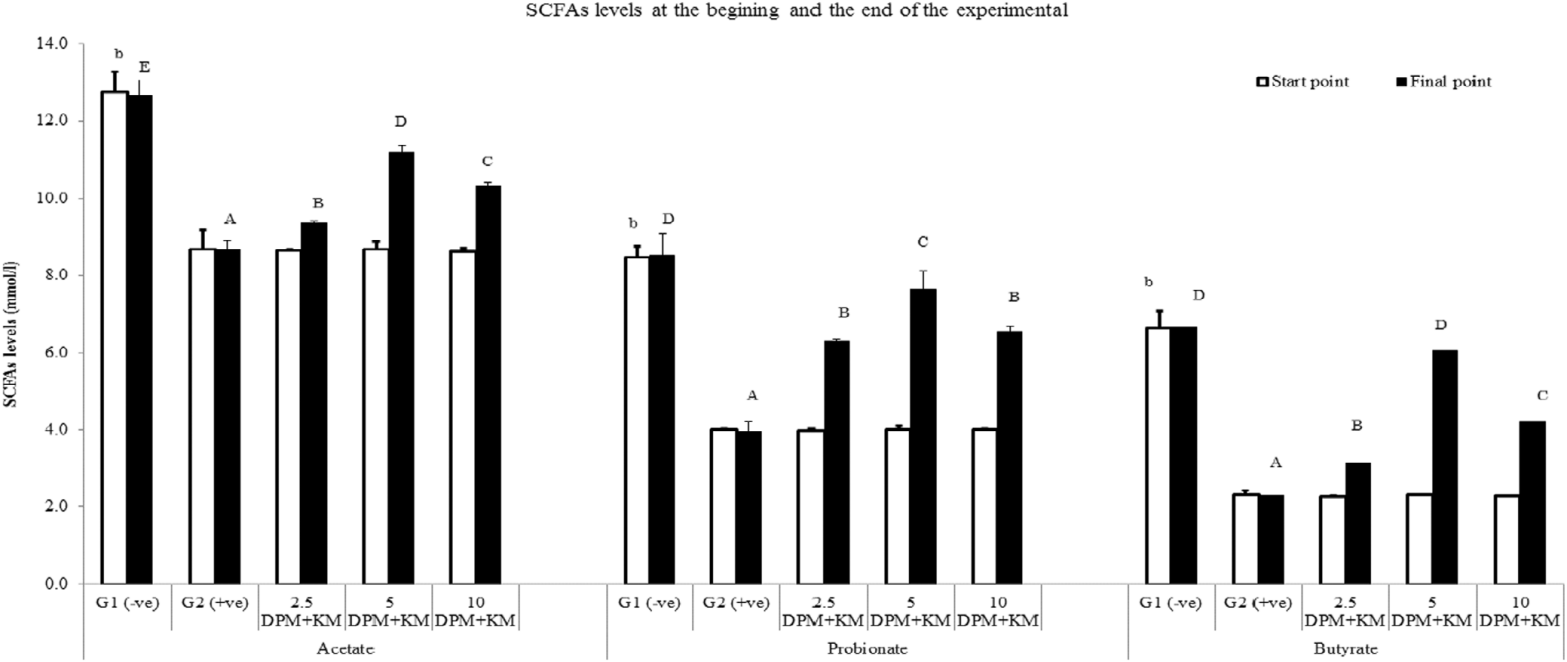
Short‐chain fatty acids (SCFAs) produced levels between hyperlipidemia rats fed dried powdered mushrooms and kefir milk (DPM/KM) supplementations at the start and the end of the experimental. Means in the different superscript small letters are significantly different (*p* ≤ 0.05) within the start time points while means with different superscript capital letters are significantly different (*p* ≤ 0.05) within the end time points between the same produced acid.

Studying the effect of using DPM with KM mixtures (DPM/KM) in different levels in association with measured SCFAs have been presented mainly for acetate, propionate, and butyrate between hyperlipidemia rat models (Figure 2). DPM with KM mixtures (DPM/KM) are used as fermentable substrates and potential prebiotic ingredients that promote colonic health in association with the colonic probiotic species.

It can be observed from Figure 2 that acetate has the highest produced levels followed by propionate and then butyrate amounts between all used animal models. The healthy control group (G1; −ve) shows the uppermost produced SCFAs levels in contrast to the hyperlipidemia animal used models that provided a normal control diet without any supplemented treatments (DPM + KM). It has presented the smallest produced beneficial SCFAs at the first time points (start time points of running the experimental; Figure 2). Also, it has been observed that the produced levels of acetate, propionate, and butyrate have been increased significantly (*p* ≤ 0.05) at the end of running the experimental (final time points), especially with the treated rats group at 5% mixture (PDM + KM) within the diet of the hyperlipidemia models. Such treated group presented recovery of all three measured SCFAs nearly to the control healthy group after the 5% mixture consumptions. While the second recovery rats group was observed within the hyperlipidemia rats fed on 10% PDM + 5% KM and that was followed by the 2.5% PDM + 5% KM additions (Figure 2).

### 3.7 The Effect of DPM/KM Supplementations on Histology Kidney Tissues (Structural Examination) Between Hyperlipidemia Animal Models

**FIGURE 3:**
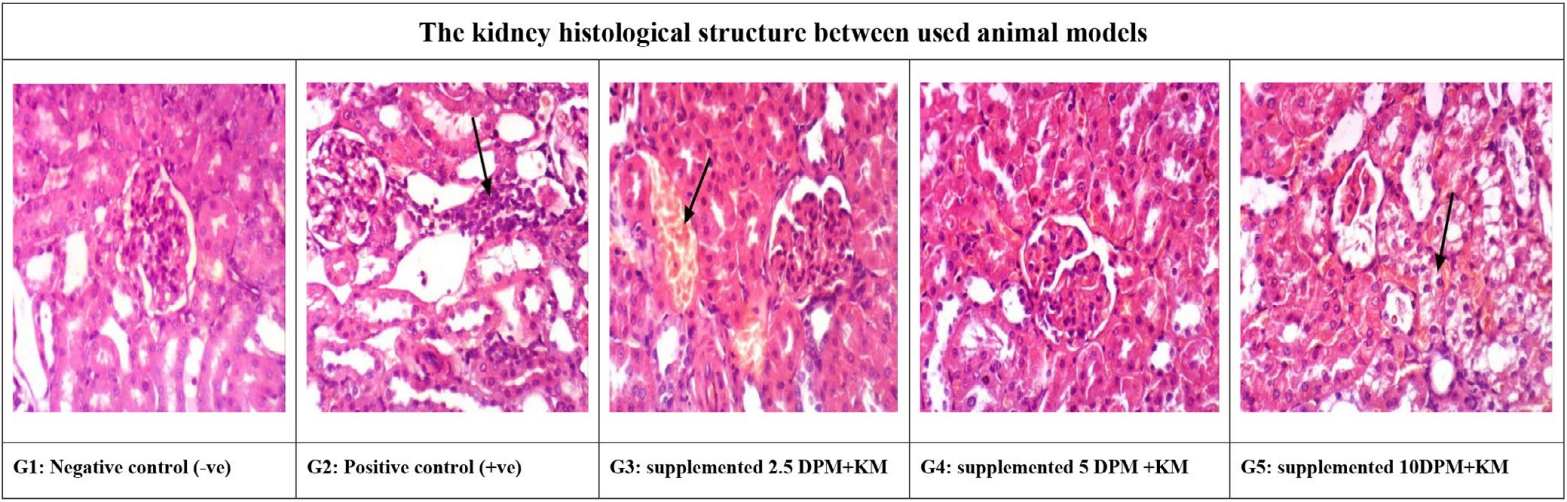
The kidney histological structure between hyperlipidemia rats fed dried powdered mushrooms and kefir milk (DPM/KM) supplementations. G1 shows healthy normal (negative control) histology of renal tissue (H&E ×400) while G2 presents the control positive group with interstitial nephritis (H&E ×400). Additionally, G3 of kidney between rats supplemented 2.5% PDM with 5% KM shows congestion of renal blood vessels (H&E ×400) and G4 is presenting and shows no histological changes with rats supplemented 5% DPM and 5% KM (H&E ×400). Finally, G5 shows the group rats fed 10% DPM and 5% KM supplementations revealed vacuolation of epithelial lining renal tubules (H&E ×400).

This study aimed to measure the effects of the used treatments on the kidney histology tissue structure. Microscopically, kidney tissues were collected from rats at G1; the control normal group (negative control; −ve) showed the normal histological structure in Figure 3A of renal parenchyma (H&E ×400). Meanwhile, kidneys of rats from G2, the control positive used rat models (+ve), showed interstitial nephritis (H&E ×400; Figure 3B). Moreover, some sections from G3 that used animal models fed 2.5% DPM and 5% KM showed congestion of renal blood vessels (H&E ×400) whereas no histological changes with rats in G4 that were on 5% DPM and 5% KM supplementations (H&E ×400; Figure 3C). Additionally other sections from G5 that were for animal models fed 10% DPM and 5% KM supplementations revealed vacuolation of epithelial lining renal tubules (H&E ×400; Figure 3).

## 4 Discussion

The hyperlipidemia condition has multi‐factorial etiology ranging from genetic and environmental factors especially dietary interactions and/or modifications such as probiotics and prebiotics supplementations. To the best of our knowledge, mushroom and kefir mixed supplementations haven't been examined in association with GM compositions and activity modulations, especially between hyperlipidemia models. The current collected data herein illustrated the hyperlipidemia *anti*‐properties of mushroom and kefir mixtures in association with GM and AI modulations between hyperlipidemia animal models. Both kefir and mushroom have been used as probiotics and prebiotics dietary sources in innovative healthy strategies through GM modulations. Mushroom is a useful food playing a vital role in human health because of low levels of polyunsaturated fat and glucose in addition to low sodium with low CHO in contrast to high antioxidant levels reducing the risk of obesity‐related dyslipidemia and hypertension (Mustafa et al. 2022). Additionally, kefir as a fermented milk product has specific bacteria and yeast combinations in different levels depending on many factors such as the type of kefir grain and milk in addition to their composition, fermentation period, and conditions of storage (Rosa et al. 2017). Also, kefir has shown many health‐promoting activities within human health in different studies but none has examined its effects when mixed with mushroom between hyperlipidemia rat models in association to GM and AI modulations. The rats fed different mixed dietary levels of both mushroom and kefir in addition to two control rat groups (−ve and +ve) were illustrated in the result sections. For example, the BW presented in Table 1 shows that the intimal BW is nearly the same (118 g) in contrast to the end of the experimental as the BW was increased with respect to the biggest BW between the control positive group (Table 1). It was followed by the rats group fed 2.5 DPM + 5% KM then 10 DPM + 5% KM and finally 5 DPM + 5% KM, respectively. Also, group rats fed 5% (DPM + KM each) and the negative control group got similar effects on the rat's BW reduction (about −33 g; Table 1). Thus both dried mushroom powder and KM consumptions in same levels show the best effective treatment on the body weigh reductions between all the hyperlipidemia rat models. Indeed kefir previous effects on body weigh changes shown declined effect. For example, a study with 12‐week kefir intervention showed slight BW reductions between MetS patients (Bellikci‐Koyu et al. 2019). Mushroom is low in calories, fats that all in contrast to high fiber and protein levels in addition to minerals and vitamins high levels resulting BW reduction and many beneficial effects on human health such as cardio‐protective, anti‐obesity and anti‐diabetic properties (Kanwal, Aliya, and Xin 2020). Indeed the data presented within the current study show that the BG levels have been decreased after the consumed treatments, especially the 5% additions of both mushroom and KM (−33%). It shows the biggest declined glucose levels nearly to the control negative group (−35%) and that support the antidiabetic effects of different previous studies. For example, mushroom supplementations between obese models reduced the body weigh in addition to the plasma glucose dropped levels (Mustafa et al. 2022). Additionally, another research shows that powdered mushroom consumption decreased the plasma total CHO levels in mice fed high fat diets (Huang et al. 2021). Indeed the consumed edible mushrooms illustrated within a recent published study with low plasma lipid profile, especially triglyceride, total CHO, and LDL‐c recommended it for obesity prevention (Mustafa et al. 2022). Regarding the AI that was previously used as a predictor of CVD risk, the levels measured here in association with the mushroom and kefir mixtures' hypolipidemia effects have been confirmed by the impaired lipid metabolism. Indeed, such a point is consistent with the collected data in the current study as the positive control group was in very high AI levels for all measured parameters as it has been illustrated in Table 4: CHO/HDL‐c, LDL‐c/HDL‐c, and CHO‐HDL‐c calculations between all groups with the biggest obtained levels at 5.85 ± 0.97, 3.52 ± 0.73 and 89.01 ± 3.13 mg/dL, respectively, in contrast to the healthy control group (G1). Such levels have been declined by the eating mushroom/kefir mixtures, especially at 5% each that were the closest to the control healthy animal models (1.46 ± 0.01, 0.27 ± 0.01 and 20.13 ± 0.33 mg/dL, respectively). It has been previously recommended that the lower atherogenic indices value is the lower CVD risk incidence (Khalil, Alfaris, and Altamimi 2022). Thus the best effective treated animal hyperlipidemia rats have been represented within the mushroom and kefir supplemented levels at 5% each within the current measured parameters. It has been shown previously that probiotics and prebiotics reduced the lipid profile levels by increasing the bile salt synthesis in addition to bile acid deconjugation that should offer the protection from CVDs incidence.

Regarding the bacterial modulations after the supplemented diets, it can be explained as previously stated that the most located bacterial species in the lower gastrointestinal tract is the pathogenic *Clostridium* that can affect the intestinal mucus if accumulated and also has been associated with gastrointestinal disorders such as ulcerative colitis (UC) and colon cancer. However, such negative conditions and effects of GM can be modulated by dietary interactions, especially with probiotics supplementations. The most commonly beneficial probiotic bacterial strains are *Bifidobacteria* and *Lactobacillus* that both can help to stimulate the strength of gut barrier functions. Baseline characteristics of the most common bacterial species in KM are *Lactobacillus*, *Streptococcus*, and acetic acid bacteria while the kefir most commonly found yeasts are *Saccharomyces* and *Candida* as mentioned in a recent kefir study and that shows GM levels, especially with the abundance of Actinobacteria, were significantly increased after daily kefir in 180 mL consumption with no significant changes in the relative abundance of *Bacteroidetes* and *Proteobacteria* to MetS patients that well known to be twice higher for CVD risk development (Bellikci‐Koyu et al. 2019). Indeed, the GM illustrated in Table 5 and Figure 1 show no differences in all measured species at the start time points between hyperlipidemia models while they have changed at the end of the experimental time points. For example, *Bifidobacteria* and *Lactobacillus* calculated species increased after PDM and KM in contrast to *Clostridium histolyticum* declined levels with respect to the mixture of 5% additions in both PDM and KM at the final time points that confirm the best prebiotic effects.

Finally, another important measured parameter is the colonic fermentation end products: SCFAs. The GM ferments food components that break down to produce such beneficial metabolites. They play a vital role in health maintenance, especially after reducing the pH within the colonic environment which in turn lowers the pathogenic bacterial growth (Hijová 2023). The produced SCFA levels mainly depend on the GM compositions; healthy and balanced colonic ecosystem produces beneficial SCFAs that then prevent difference related gastrointestinal diseases such as UC, diabetes, and obesity as shown within our previous published data (Khalil et al. 2014, 2021). Acetate and propionate in addition to butyrate are the main SCFAs in the gut formed by GM in approximately 80% of all SCFAs. They are presented in the colon and feces at about 60:20:20 M ratio each for acetate, propionate, and butyrate, respectively. However, such presented levels can be affected by different conditions such as the microbiota composition, gut transit time, and available fermentable substrates (Hijová 2023). Indeed the current collected measured SCFA levels between hyperlipidemia rats model showed the best compromised levels at the probiotic and prebiotic substrate available (5% supplemented mushroom and kefir levels) in association to the GM compositions (Bif and Lab in contrast to His levels). Thus the current study has shown the best effective innovative strategy for CVD preventions may be due to the equality consumed probiotics and prebiotics supplementations. However, much more human and supplemented levels for the long term are needed.

Microscopically, tissues collected from the kidney of used rats have been measured as G1, which was for the control normal group (negative control; −ve) that shows normal histological structure as recorded in Figure 3 of renal parenchyma (H&E ×400). Also, kidneys of G2 rats for the control positive used rat models (+ve) demonstrated interstitial nephritis (H&E ×400; Figure 3). Moreover, some sections of rats in G3 group that used animal models fed mixture of 2.5% DPM and 5% KM show congestion of renal blood vessel (H&E ×400). While no histological changes with rats in G4 fed on 5% DPM mixed with 5% KM supplementations (H&E ×400; Figure 3). Additionally other sections from G5 that were for animal models fed 10% DPM mixed with 5% KM supplementations revealed vacuolation of epithelial lining renal tubules (H&E ×400; Figure 3). The current study is in accord with the positive impact on kidney examinations after the supplemented DPM mixed with 5% KM supplementations. It shows protective roles on renal function among hyperlipidemic animal models used especially between high supplemented levels thus improving all effects and prevents degenerative changes of kidney tissues with no histological changes as in control rats (Qin et al. 2019).

## 5 Conclusion

To conclude, the hyperlipidemia condition within the used current dietary supplementations as functional synbiotic (probiotics and prebiotics) dietary sources with respect to mushroom and kefir mixed supplementations between rat models presented the best effective supplemented levels at 1:1 percentage (refers to 5% DPM and 5% KM). Illustrated results persuaded mushroom and kefir mixtures have improved and controlled the hyperlipidemia models' BW, BG, lipid profile with the AI, and finally the colonic microbiota compositions and activities that should be considered within further human study. All results have proven that mushroom and KM reduced the model's BW, BG, and lipid profile. Also, GM increased within the beneficial species (*Bifidobacteria* and *Lactobacillus*) in contrast to *Clostridium histolyticum* reduction numbers in association with the SCFAs. The healthy conditions within the hyperlipidemia group fed 5% of both PDM + KM supplementations improved; however, much more human studies in correlation to GM alterations are needed.

## Author Contributions

**Huda Aljumayi:** validation (equal), visualization (equal), writing – review and editing (equal). **Amani A. Alrasheedi:** investigation (equal), visualization (equal), writing – review and editing (equal). **Thamer Aljutaily:** methodology (equal), writing – review and editing (equal). **Isam A. Mohamed Ahmed:** resources (equal), validation (equal), writing – review and editing (equal). **Nazeha A. Khalil:** conceptualization (equal), formal analysis (equal), methodology (equal), visualization (equal), writing – review and editing (equal).

## Ethics Statement

The current study has been approved ethically and biologically by the academic professional scientific research ethics committee at the Nutrition and Food Sciences Department, Menoufia University, Egypt, under the safety and well‐being conditions (Animal Care and Use: 16‐SREC‐04‐2023).

## Conflicts of Interest

The authors declare no conflicts of interest.

## Data Availability

The data supporting the conclusions of this article are included in the manuscript.
